# Supplementary material for: Efficient population size of urban agglomerations in the Yangtze River Economic Belt from a financial perspective
Source: PLoS One. 2024 Sep 27;19(9):e0311090. doi: 10.1371/journal.pone.0311090 (PMC11432864; doi:10.1371/journal.pone.0311090)
Supplement: Attachment — Submitted filename: Response to Reviewers.docx [file pone.0311090.s002.docx]

**Response to review comments**

**Response to reviewer #1:**

The paper can be accepted after making the following minor revisions.

1. The innovation of this paper needs to be highlighted in the abstract.

Add nine lines of text at the end of the abstract to introduce the innovation of this article ((Line 28-Line 36).

2. The article lacks an important discussion link, in which the author should focus on describing the differences between the article study and other scholars' studies, thus highlighting the relevance and academic value of the article, the following literature should be helpful for your research: (1) Reduction pathways identification of Agricultural Water Pollution in Hubei Province, China. (2) A differential game of water pollution management in the trans-jurisdictional river basin. (3) Coordination of the Industrial-Ecological Economy in the Yangtze River Economic Belt, China.

Thank you for your valuable suggestion. We have added a discussion section (Line 424-Line 468). However, due to the differences between these three references and the main topic of this study, a comparative citation was not made in the discussion section.

3. The article was not written following the correct journal's guidelines to be considered for publication. INTORDCUTION→MRTHOD→RESULTS→DISSCUSION→CONCLUSION

The revised article has been restructured and laid out according to the correct journal guidelines.

4. How can your study inspire studies on other regions? The results should be further elaborated to show how they could be used for the real applications.

Set up "Conclusion" section (5.1) at the end of the article to introduce how the study inspire studies on other regions and how it be used for the real applications.

5. What are the managerial insights/policy implications of this study? Compared with available literature, what are the theoretical contributions and application values of this study? It is suggested to enhance the corresponding discussions in the conclusion part.

Set up "Conclusion" section (5.2) at the end of the article to introduce the managerial insights/policy implications of this study，or the theoretical contributions and application values of this study.

**Response to reviewer #2:**

The study quantified the effective population size of three urban agglomerations from a financial perspective. By reading the manuscript, I have the following comments and questions:

1. Line 57 of the manuscript fails to provide a detailed elaboration of the universal laws mentioned therein. Furthermore, the text refers to "Western researchers," yet the subsequent cases presented are all Chinese scholars and studies.

This study did not express it clearly. In fact, it is to say that these laws also exist in the development process of urban agglomerations in China. Therefore, the examples given later are all studies by Chinese scholars.

By adding the wording of “These general laws discovered by Western scholars also exist in the development process of urban agglomerations in China.” (Line 77-Line 78，Red font section), can solve this problem.

2. The introductory section of the manuscript provides a comprehensive overview of existing studies, yet lacks the requisite summaries and generalisations. Furthermore, the necessity, importance, innovativeness, and significance of the study are not adequately addressed in the introductory section.

Delete Lines 99-Lines 102 (Text before modification) in the introductory section. And add the requisite summaries and generalisations in the introductory section (Line 120-Line 139, the blue).

Add content on the necessity and importance of the study (Line 59-Line 70).

Add content on the innovation and significance of the study (Line 140-Line 148).

3. It is recommended that the reasons for the selection of the study area be included in the manuscript. Furthermore, it is advised that the authors provide an overview map of the study area, indicating the location of the cities and their spatial extent.

Added an overview map of the study area (Fig. 1 in the revised article).

4. Line 176-Line 183: This section of the statement appears superfluous and lacks any meaningful connection to the study.

After consideration, this section only introduces the four main typical indicators for evaluating the risk of fiscal deficits and does not have much practical significance. So delete Line 176-Line 187 (Text before modification), and delete the reference [13] (Text before modification).

5. The figures and their accompanying labels in the manuscript are too small to facilitate the acquisition of the requisite information. Furthermore, the names of the urban agglomerations are incorrectly labelled. It is recommended that the authors incorporate the contents of Figures 2, 3, and 4 into Figure 1 in order to enhance the visual presentation of the results.It is recommended that the authors provide the goodness of fit in Figure 5.

Provide original Figures to solve the problems of the figures and their accompanying labels in the manuscript are too small to facilitate the acquisition of the requisite information.

The incorrect names of the urban agglomerations in the relevant figures have been corrected, and all names of the urban agglomerations in the previous and subsequent sections have been reconfirmed.

Incorporate the contents of Figures 2, 3, and 4(and delete Figures 2, 3, 4, Part of the content is placed in Line 263-Line 267) into Figure 1(Figure 2 in the revised article) in order to enhance the visual presentation of the results.

Provide the goodness of fit in Figure 5 (Figure 3 in the revised article), and provide original Figures to solve the problems of the figures and their accompanying labels in the manuscript are too small to facilitate the acquisition of the requisite information.

6. The Conclusions and Discussion section, which is largely identical to the results, should be summarized as part of the conclusions. Furthermore, the extended discussion and analysis of the results in the existing manuscript is inadequate to highlight the value of the study.

Thank you for your valuable suggestion. We have added a discussion section (Line 424-Line 468). And the research value of this study was explained in the conclusion section.

7. Furthermore, the outcomes of linear/non-linear fitting based on a single factor are frequently characterised by uncertainty and ambiguity. It is therefore recommended that authors consider a multifactor combined analysis in order to enhance the accuracy and representativeness of the results.

Thank you for your suggestions on the methods presented in this study. In order to clearly examine the relationship between population size and fiscal revenue / expenditure in the three urban agglomerations along the Yangtze River Economic Belt, as well as the trend changes of this relationship between years, this study uses the linear/non-linear fitting based on a single factor. Of course, multifactor combined analysis can more accurately simulate the numerical impact of this relationship, but at the same time, the intervention of other factors may lead to the emergence of multiple effects between variables, which is not conducive to the realization of the purpose of this article. Japanese scholars such as Mori have also used this method to study similar issues in the Tokyo metropolitan area and Nagoya metropolitan area. Therefore, it is currently not suitable to make modifications to this article based on your suggestions. In the future, our research team will conduct further research based on your insightful suggestions to compensate for the lack of research in this field.

In summary, it is recommended that the manuscript be given a major revision.
