# Supplementary material for: Efficient population size of urban agglomerations in the Yangtze River Economic Belt from a financial perspective
Source: PLoS One. 2024 Sep 27;19(9):e0311090. doi: 10.1371/journal.pone.0311090 (PMC11432864; doi:10.1371/journal.pone.0311090)
Supplement: Attachment — Submitted filename: Response to Reviewers Comments.docx [file pone.0311090.s003.docx]

**Reviewer Comments:**

1. The author's correspondence email address should be an institutional email address. Please try to use the university official email instead of Outlook email.

Answer: I have changed the author's correspondence email address to the school's official email address and made sure I no longer use Outlook email. Thank you, sir.

2. Abstract is very boring. Please hit the pillars of academic abstract (i.e., background information, methods, important results, implications in brief). The current abstract lacks background information, the methods are not clearly presented, and the results and significance are too lengthy.

Answer: I have re-written the abstract and structured it around background information, methodology, key findings, and brief meaning to make it more attractive and informative. Thank you, sir.

Urban agglomerations, such as the Yangtze River Delta, Yangtze River Middle Reaches, and Chengdu–Chongqing regions, play a crucial role in driving China's regional economic development. While previous studies have focused on economic and social aspects, the fiscal dimension of urban agglomerations remains underexplored. This study addresses this gap by investigating the relationship between population size and fiscal efficiency in these three major urban agglomerations along the Yangtze River Economic Belt (YREB).We introduce the concept of fiscal efficiency based on revenue and expenditure and select relevant indices, such as efficient population size and fiscal self-reliance. Using statistical data from 2017 to 2019, we employ curve regression analysis in SPSS to estimate the efficient population sizes of these urban agglomerations and examine differences in financial efficiency over time and space.Our analysis reveals that cities with populations over 10 million hinder fiscal efficiency in the Yangtze River Delta, those with 3–5 and 5–10 million in the Yangtze River Middle Reaches, and those with 5–10 and 1–5 million in the Chengdu–Chongqing urban agglomerations. The maximum financially efficient population sizes are estimated at 648 million for the Yangtze River Delta, 308 million for the Yangtze River Middle Reaches, and 320 million for the Chengdu–Chongqing urban agglomerations. Considering various fiscal indicators, all three agglomerations demonstrate varying degrees of efficiency.The innovation of this study lies in the interdisciplinary approach, integrating finance, demography, urban planning, and regional economics. By analyzing population size from a fiscal perspective, we provide a novel theoretical framework and analytical tool for policymakers. This study highlights the importance of fiscal balance and population optimization in urban agglomerations, contributing to regional coordinated development and sustainable growth.

3. Introduction: Please don't start by arguing about China. This is an international journal. Please start by giving some background information suitable for international readers. The format of "Wang et al. [5] - [8]" is incorrect.

Answer: I have revised the introduction to remove the phrase that begins with the Chinese debate and to add background information for an international audience. Also, the citation format of "Wang et al. [5] - [8]" has been corrected. Thank you, sir.

Urban agglomeration represents the advanced stage of urban development, characterized by the clustering of cities into highly integrated systems. This phenomenon is not only prevalent in developed countries but also increasingly significant in rapidly developing nations like China. The Chinese government has identified urban agglomerations as pivotal to regional development and has outlined strategies to foster their growth, particularly in key areas such as the Beijing–Tianjin–Hebei region, the Yangtze River Delta, the Guangdong–Hong Kong–Macao Greater Bay Area, the Chengdu–Chongqing region, the Yangtze River Middle Reaches, the Central Plains, and the Guanzhong Plain. Despite achieving an urbanization rate exceeding 60% by the end of 2020, there is a consensus that China's urbanization should ideally reach around 75%, considering the proportion of rural labor. The ongoing migration of populations from third- and fourth-tier cities to central urban areas is amplifying the effects of urban agglomeration, driving China's economic development. However, this rapid urbanization also brings challenges, including discrepancies in development needs and concepts across different regions. Against this backdrop, the study of urban agglomerations, particularly their effective population size, becomes crucial for understanding and addressing these issues.

4. The introduction section should end with a paragraph describing the organization of the subsequent sections. The research processes for this manuscript should be given (see Figure 1 of the reference given below).

Answer: I have added a paragraph at the end of the Introduction describing the organization of the subsequent chapters and outlining the process of research into this manuscript. Thank you, sir.

The research process of this paper is shown in Figure 1.This study focuses on the effective population size of the three major urban agglomerations along the Yangtze River Economic Belt (YREB): the Yangtze River Delta, the Yangtze River Middle Reaches, and the Chengdu–Chongqing urban agglomerations. We adopt a fiscal perspective, recognizing the significant role finance plays in shaping urban agglomerations and influencing government decisions. Previous studies have primarily examined urban agglomerations from economic and social viewpoints, with less attention paid to the fiscal implications. To address this gap, we will analyze the relationship between population size and fiscal efficiency, aiming to provide a more comprehensive understanding for policymakers. The methodology involves a detailed review of existing literature, quantitative analysis of population and fiscal data, and comparative case studies. The remainder of this paper is organized as follows: Section 2 provides a detailed literature review, Section 3 outlines the methodology and data sources, Section 4 presents the results and discussion, and Section 5 concludes with policy implications and suggestions for future research.

Figure 1 Research process

5. Some sentences are so strange that the authors need to find a professional agency to proofread them. For example, Line 187, "The data required for this study involve the populations of urban agglomerations, fiscal revenues, and fiscal expenditures, among other data.", What do you mean by among other data? The figure of the study area needs to be redrawn, and the source and type of data need to be organized in tables (see Table 1 in reference attached below).

Answer: I have contacted a professional body to proofread the article and to revise the sentence on line 187. At the same time, I'll redraw the graph of the study area and organize the source and type of data in tabular form. Thank you, sir.

6. Line 208: Based on Nakamura and Kanauchi’s [12], This way of writing is wrong.

Answer: I have removed the incorrect quotation in line 208 and corrected it to the correct format. Thank you, sir.

7. In equations 1 and 2, all parameters/variables (i.e., N, Y, C and E) need to be italicized. Meanwhile, I don't understand the meaning of functions f1 and f2. Line 215: "By deriving N in Equation (2), the maximum value of Y/C can be calculated." I find it hard to understand what this sentence means.

Answer: I have italicized the arguments/variables in Equations 1 and 2 and explained the meaning of the functions f1 and f2 in detail. At the same time, I rephrased the sentence on line 215 to improve clarity. Thank you, sir.

8. Line 254: R2 should be deleted.

Answer: I have removed R2 on line 254. Thank you, sir.

9. Line 301: There is a problem with the format of the formula. All equations in the manuscript should be edited using software such as Mathtype.

Answer: I have edited all the equations in the article using software such as Mathtype to make sure they are formatted correctly. Thank you, sir.

10. Discussion section should be improved. It should present core results that emerge from the study, interprets and discusses them (by the authors). The discussion section needs to compare this study with other key studies, emphasize the significance of this study, provide implication for academia (theoretical) and policy and decision makers (managerial) (what benefit should be gained out of such result, what does it mean to port managers and policy makers). If I read the study, what is the benefit I gain as new thing? Readers, researchers and policy makers, should leave with a bunch relevant implication not only results. Some of the management insights given so far are so obvious that they can be obtained even without conducting this research. For the specific writing method of this section, the author can refer to the reference attached below.

Answer: I have refined the discussion section to highlight the core findings of the study, explain and discuss those findings, and compare this study to other key studies to highlight the significance of this study and provide inspiration to academia and policymakers. Thank you, sir.

At present, research on urban agglomerations mostly stays at the level of review and qualitative description, with less empirical research and a lack of comparative analysis of detailed spatiotemporal data. Existing research mainly focuses on urban agglomerations in the northeast of the United States, Pacific coastal urban agglomerations in Japan, London urban agglomerations in the United Kingdom, and northwestern European urban agglomerations. For China's urban agglomerations, which are still undergoing industrial transformation, the trend of population aggregation within urban agglomerations towards central cities continues. However, the large outflow of population due to insufficient economic development vitality has become an important factor affecting regional coordinated development, and serious problems such as the lack of effective coordination between central cities and other small and medium-sized cities in urban agglomerations have also emerged. From the perspective of urban agglomeration development, there are still a few mature urban agglomerations among the 19 major urban agglomerations in China. Most urban agglomerations are undergoing a critical stage of transformation, and population and financial development have not yet formed a good collaborative relationship, such as the Harbin Great Wall urban agglomeration, the Liaozhong South urban agglomeration, the Yangtze River middle reaches urban agglomeration, and the Chengdu Chongqing urban agglomeration [19]. Among the three urban agglomerations discussed in this study, only the Yangtze River Delta urban agglomeration has formed a good cooperation relationship between population and finance.

Zipf [20] pioneered the introduction of the law of universal gravitation in physics to analyze the spatial structure of urban agglomerations. Subsequently, empirical research on the spatial structure of urban agglomerations flourished, and scholars studied the spatial structure of urban agglomerations from multiple perspectives. Friedmann [21] simulated the stages and processes of spatial structure evolution in urban agglomerations based on the "Core-Edge" theory. Yu [22] studied the spatial structure evolution process and characteristics of the Beijing metropolitan area by constructing regional space. Chin [23] constructed a statistical model on population employment and commuting data to empirically study the spatial structure of metropolitan areas in the Midwest of the United States. Tsai [24], Meijers and Burger [25] describe spatial structure from three aspects: scale dimension, single center - multi center dimension, and agglomeration - diffusion dimension. The innovation in methodology of this study is mainly reflected in the introduction of a fiscal perspective, the combination analysis of efficiency and population size, multidimensional data analysis, innovative model construction, and the combination of dynamic and static analysis. The use of these innovative methods can more comprehensively reveal the relationship between population size and fiscal efficiency in the national level urban agglomeration of the Yangtze River Economic Belt, and make up for the shortcomings of existing research from a new perspective.

Discussion on the stages of population spatial distribution in urban agglomerations. The "Spatial Cycle Hypothesis" suggests that the population spatial structure of urban agglomerations will undergo processes of urbanization, suburbanization, reverse urbanization, and re urbanization [26]. There are also studies suggesting that the population spatial distribution of urban agglomerations will undergo three stages: linear decline, inverted U-shaped distribution, and "M-shaped" distribution [27]. This study found that the population spatial distribution of the Yangtze River Delta urban agglomeration, the middle reaches of the Yangtze River urban agglomeration, and the Chengdu Chongqing urban agglomeration showed different, such as exponential increase, U-shaped distribution, and inverted U-shaped distribution, respectively. And filling the gap in existing research on population spatial distribution in urban agglomerations.

Recent studies have explored various aspects of socio-economic development and environmental impacts in the Yangtze River Economic Belt (YREB). Cai and Tu [28] analyzed the spatiotemporal characteristics and driving forces behind construction land expansion in the YREB, highlighting the complex dynamics shaping urban growth. Chen and Shen [29] examined the spatiotemporal differentiation of urban-rural income disparity and its driving forces between 2000 and 2017, providing insights into regional inequality. Li, Chai, and Ren [30] investigated the spatial spillover effects of urban innovation on productivity growth across 108 cities in the YREB, underscoring the role of innovation in regional development. Tian, Huang, and Zhao [31] conducted a comprehensive study on green innovation, including spatial analysis, coupling coordination, and efficiency evaluation, which is crucial for sustainable development in the region. Lastly, Wang, Zhao, and Wang [32] employed random forest analysis to identify factors affecting urban carbon emissions within the YREB, emphasizing the need for targeted policies to mitigate environmental impacts.

11. The conclusion should be organized better. That is, provide a summary of the study, brief results, in addition to proper limitations and future research areas. The conclusion currently lacks the simple introduction and specific conclusions of this article. For the specific writing method of this section, the author can refer to the reference attached below.

Answer: I have reorganized the conclusion section to provide a summary of the study, brief the results, and indicate appropriate limitations and areas for future research. Thank you, sir.

This study investigates the efficient population sizes of key urban agglomerations in the Yangtze River Economic Belt (YREB) from a fiscal perspective. By analyzing the relationship between population size and fiscal efficiency, we identify the maximum financially efficient population sizes for the Yangtze River Delta, Yangtze River Middle Reaches, and Chengdu–Chongqing urban agglomerations. Our findings reveal that the optimal population sizes for fiscal efficiency vary across these regions, with specific thresholds for different city sizes acting as critical factors.

The core results of this study highlight the importance of balancing population size and fiscal efficiency in urban agglomerations. For the Yangtze River Delta, cities with populations over 10 million are a focal point for improvement. In the Yangtze River Middle Reaches, cities with populations of 3–5 million and 5–10 million need targeted strategies. Similarly, for the Chengdu–Chongqing urban agglomeration, cities with populations of 5–10 million and 1–5 million require specific attention. These insights are crucial for policymakers aiming to optimize urban development and resource allocation.

Our research contributes to the field by providing a quantitative framework for analyzing the efficient population sizes of urban agglomerations. This framework complements existing theories and empirical studies by incorporating a fiscal perspective. Moreover, the practical implications of our findings offer guidance for policymakers and urban planners on how to manage urban growth and optimize fiscal resources effectively.

In summary, this study advances our understanding of the complex interactions between population size and fiscal efficiency in urban agglomerations. It underscores the need for targeted policies that consider both the size and fiscal health of cities within these regions. By doing so, it provides a roadmap for fostering sustainable, healthy, and coordinated development in the YREB and other similar urban agglomerations.

This study not only delves into the issue of effective population size in the Yangtze River Economic Belt urban agglomeration, but also provides a new perspective for understanding the relationship between urban development and population size from a fiscal perspective. For research in other regions, this article provides valuable insights in multiple aspects and can further refine the results to guide practical applications.

5.1. Theoretical contributions and inspirations

This article combines knowledge from multiple disciplines such as finance, urban planning, and demography, providing a comprehensive perspective for understanding the development of urban agglomerations. This inspires researchers in other regions to cross the boundaries of traditional disciplines and comprehensively utilize multidisciplinary knowledge and methods when conducting similar research, in order to obtain more comprehensive and in-depth analysis results. From a fiscal perspective, the impact of population size on fiscal revenue and expenditure, public service provision, and other aspects was analyzed. This reminds researchers in other regions that when exploring population size issues, in addition to considering conventional factors such as economy and society, they should also pay attention to the influence of fiscal factors to comprehensively evaluate the rationality of population size.

5.2 .Policy implications and application values

The practical application areas based on the research results of this article are: (1) Local governments can formulate more precise population policies. For example, in areas with high population inflows, the government can increase investment in public services and improve urban carrying capacity. In areas with high population outflow, the structure of fiscal expenditure can be optimized and the efficiency of fiscal fund utilization can be improved. (2) The urban planning department can draw on the ideas of this study and develop reasonable urban planning plans based on local conditions. By scientifically predicting population size, rational layout of urban space and infrastructure, ensure that urban development is coordinated with population size. (3) According to the trend of population size changes, the government can adjust its public service provision strategy. For example, in the fields of public services such as education, healthcare, and transportation, resources should be allocated reasonably based on changes in population demand to improve the quality and efficiency of public services. (4) Establish a long-term population size monitoring mechanism, regularly collect and analyze relevant data, and timely grasp the trend of population size changes. By comparing and analyzing data from different regions and time periods, provide scientific basis for policy formulation and planning. (5) Establish a population size evaluation system and regularly evaluate the effectiveness of policies. By evaluating the results, adjust policy direction and measures in a timely manner to ensure the achievement of policy objectives.

5.3. Research Outlook

(1) Focus on improving the methods of data collection and processing to enhance the accuracy and completeness of data. At the same time, unified data collection and statistical standards should be established to better compare and analyze data from different cities and regions. (2) Existing models and methods should be further improved and expanded to comprehensively consider various factors that affect the effective population size. Especially, the analysis of complex factor interactions should be strengthened to provide more accurate and in-depth insights. (3) In addition to the fiscal perspective, future research should also be comprehensively analyzed from multiple perspectives such as society, economy, and environment. This will help to comprehensively evaluate the development status of urban agglomerations and provide a more comprehensive and scientific basis for policy formulation and planning. (4) More attention should be paid to the role of regional and cultural differences in population size and its impact. By conducting in-depth research on population size changes and their causes in different regions and cultural backgrounds, more specific and targeted recommendations can be provided for the development of urban agglomerations.

12. The formatting of the references in this manuscript needs revision. I often see inappropriate citations. The author should also cite some articles in this journal.

Answer: I have cited more references from the magazine.

13. The number of cited literature is relatively small (only 27 literature), which is also related to the insufficient literature review. I suggest that the author strengthen his/her review of existing literature in the introduction section.

Answer: I have revised the format of the references and increased the number of citations, as well as a more comprehensive review of the existing literature. Thank you, sir.

In addition to the problems I pointed out, the author also needs to carefully sort out the flow of the manuscript and try to make the manuscript clear and fluent. At the same time, I think the format of the following article is more reasonable for the author's reference.

Reference:

Zhang, T., Xin, X., He, F., Wang, X., & Chen, K. (2023). How to promote sustainable land use in Hangzhou Bay, China? A decision framework based on fuzzy multiobjective optimization and spatial simulation. Journal of Cleaner Production, 414, 137576.

Answer: I have revised References according your comments.
